# MicroRNA-138 Overexpression Alters Aβ42 Levels and Behavior in Wildtype Mice

**DOI:** 10.3389/fnins.2020.591138

**Published:** 2021-01-14

**Authors:** Emmanuelle Boscher, Claudia Goupil, Serena Petry, Rémi Keraudren, Andréanne Loiselle, Emmanuel Planel, Sébastien S. Hébert

**Affiliations:** ^1^Centre de Recherche du CHU de Québec – Université Laval, CHUL, Axe Neurosciences, Quebec City, QC, Canada; ^2^Faculté de Médecine, Département de Psychiatrie et de Neurosciences, Université Laval, Quebec City, QC, Canada

**Keywords:** Alzheimer’s disease, microRNA, MiR-138, memory, anxiety, adeno-associated virus

## Abstract

Alzheimer’s disease (AD) is a progressive neurodegenerative disorder characterized by changes in cognitive and behavioral functions. With the exception or rare mutations in PSEN and APP genes causing early-onset autosomal dominant AD (EOADAD), little is known about the genetic factors that underlie the vast majority (>95%) of early onset AD (EOAD) cases. We have previously identified copy number variations (CNVs) in microRNA genes in patients with EOAD, including a duplication of the MIR-138-2 gene. Overexpression of miR-138 in cultured cells increased Aβ production and tau phosphorylation, similar to what is seen in AD brain. In this study, we sought to determine if miR-138 overexpression could recapitulate certain features of disease *in vivo* in non-transgenic mice. A mild overexpression of pre-miR-138 in the brain of C57BL/6J wildtype mice altered learning and memory in a novel object recognition test and in the Barnes Maze. Increased levels of anxiety were also observed in the open-field test. MiR-138 upregulation *in vivo* caused an increase in endogenous Aβ42 production as well as changes in synaptic and inflammation markers. Tau expression was significantly lower with no overt effects on phosphorylation. We finally observed that Sirt1, a direct target of miR-138 involved in Aβ production, learning and memory as well as anxiety, is decreased following miR-138 overexpression. In sum, this study further strengthens a role for increased gene dosage of MIR-138-2 gene in modulating AD risk, possibly by acting on different biological pathways. Further studies will be required to better understand the role of CNVs in microRNA genes in AD and related neurodegenerative disorders.

## Introduction

Alzheimer’s disease (AD) is a neurodegenerative disorder characterized by amyloid (senile) plaques composed of amyloid-beta (Aβ) peptides and neurofibrillary tangles composed of hyperphosphorylated tau protein (Brion et al., 1985; Murphy and LeVine, 2010). Clinical traits associated with AD include progressive cognitive decline and behavioral alterations, including impaired memory and increased symptoms of anxiety (Van Dam et al., 2016). The disease can be divided into early-onset AD (EOAD) (<65 years) and late-onset AD (LOAD) (>65 years). Interestingly, the genetic contribution of LOAD is estimated to be ∼70%–80% while in EOAD this number can reach up to 100% (Gatz et al., 2006). However, only autosomal dominant mutations in *APP*, *PSEN1*, or *PSEN2* genes are identified as causal in a small fraction of EOAD cases (1%–5%) (Campion et al., 1999; Bertram and Tanzi, 2005). Thus, the vast majority of EOAD seems related to other genetic factors or mutations, possibly in both coding and non-coding regions (Prendecki and Dorszewska, 2014; Ghanbari et al., 2016; Bellenguez et al., 2017; Ludwig et al., 2019).

Abnormal gene dosage effects have been associated with AD development. The strongest evidence comes from copy number variations (CNVs) of the *APP* gene that causes autosomal-dominant EOAD (Rovelet-Lecrux et al., 2006). Other CNVs associated with disease risk include *CR1* (Brouwers et al., 2012), *CREB1* (Li et al., 2012), olfactory receptors (Shaw et al., 2011), and *BIN* (Szigeti et al., 2013) genes. Interestingly, CNVs of microRNA (miR) genes have been identified in neurological disorders, including schizophrenia (Warnica et al., 2015), autism (Vaishnavi et al., 2013), and intellectual disability (Qiao et al., 2013). Recently, we identified CNVs of miR genes in EOAD, including a duplication of the MIR-138-2 locus (Boscher et al., 2019).

The MIR-138-2 gene is highly conserved and expressed in the brain (Obernosterer et al., 2006). A functional role for MIR-138-1 specifically in Schwann cells has also been proposed (Lin et al., 2018). MiR-138 can participate in dendritic spine morphogenesis and axonal regeneration *in vitro* (Siegel et al., 2009; Sullivan et al., 2018), while changes in miR-138 expression levels are linked to memory (Schröder et al., 2014; Li et al., 2018) and anxiety (Muiños-Gimeno et al., 2011) in mice. Furthermore, Schröder et al. (2014) identified a polymorphism near the MIR-138-1 gene associated with memory performance in humans. Interestingly, miR-138 was shown by us and others to regulate tau phosphorylation in cells (Wang X. et al., 2015; Boscher et al., 2019). In addition, miR-138 was shown to be upregulated in the CSF of AD patients (Cogswell et al., 2008). Conversely, circHDAC9, a circular RNA able to repress miR-138 expression, is reduced in AD CSF (Lu et al., 2019). Finally, miR-138 can promote Aβ production in an AD mouse model and cells (Boscher et al., 2019; Lu et al., 2019). All these observations led us to investigate the biological effects of MIR-138-2 overexpression on behavior and endogenous AD biomarkers in wildtype mice.

## Materials and Methods

### Viruses

AAV2 viruses were produced by the CERVO Brain Research Center^1^. To reproduce increased MIR-138-2 dosage, the pre-miR-138-2 sequence was retrieved from UCSC Genome Browser^2^, amplified by PCR, then inserted into pcDNA plasmid followed by the AAV2/DJ8-CAG-eGFP adenovirus plasmid (Supplementary Figure S1). The amplified sequence corresponded to 150 basepairs upstream and downstream of the mouse MIR-138-2 precursor sequence and cloned downstream of the CAG promoter and eGFP coding sequence. Probes used for cloning were: 5′-cagctttctagaggtggctaaacagttagtctaccc and 5′-cgatctctagaagagctccatggacagagatgtct. Note that pre-miR-138 mouse sequence is 100% identical to the human sequence, albeit the human sequence is 12 bp longer according to miRbase.org (Supplementary Figure S2). The mature miR-138-5p sequence is fully conserved.

### Mice

C57BL/6J breeding pairs were purchased from Jackson Laboratory^3^ to generate experimental mice (*n* = 13 males and *n* = 17 females). A total of 30 littermate mice were injected at P0 with AAV2/DJ8-CAG-eGFP-MIR-138-2 (*n* = 14, miR-138) or AAV2/DJ8-CAG-eGFP (*n* = 16, control, Ctrl) which have a neuronal serotype (Hammond et al., 2017). Injections were performed with 33 gauges Hamilton syringe, at 0.25 mm laterally from sagittal suture, 0.50 mm rostral from coronary suture, and at 3 mm of deep with mean speed of 1 μl/s (Glascock et al., 2011). Injections were performed at P0 by intracerebroventricular and bilaterally manner, with 10^10^ of viral charge by hemisphere. Mice were cryo-anesthetized for 2 min before injections. The syringe was removed 2 min after injection to avoid liquid reflux. Mice were placed on a heating mat until they woke-up and then returned to their mother. Three mice per group were sacrificed 2 months after injection to study the localization of AAV-eGFP expression. Other mice were sacrificed 4 months after injection for cognitive and behavioral testing as well as biochemistry studies. All mice were sacrificed by decapitation and the brain were removed, dissected on ice, and frozen on dry ice, as previously described (Hernandez-Rapp et al., 2016). Tissues were directly stored at −80°C until use for biochemistry or fixed with 4% paraformaldehyde for immunofluorescence. All mouse studies were performed in accordance with the Université Laval ethics guidelines and regulations and approved by the VRRC Comité de protection des animaux committee.

### Open Field

After 30 min of acclimation to the testing room, each mouse was placed in the center of a white box (33 cm^2^) during 10 min allowing them to explore freely. Lux intensity was homogenous (300 lux). The box was virtually divided in center and four corners and the movements were recorded. Locomotor activity and anxiety-like behavior were evaluated through general locomotor ability and movement in the center compared to corners, respectively. Speed, distance traveled, immobility duration, and the number of entries and time passed in center and corners were analyzed with the Any maze browser (Stoelting Co, Wood Dale, IL, United States). After each test, boxes were cleaned and dried. All tests were done blind.

### Novel Object Recognition

Mice were familiarized to a white box (40 cm × 20 cm × 40 cm) for 10 min prior to testing. These boxes were lighted by homogeneous light (300 lux). During training, mice could explore two identical objects during 10 min. The next day, during the retention test; one of the objects was replaced with a new object. Mice were allowed to explore the familiar object and the novel object during 5 min. Exploration was scored when the head of the mouse was oriented toward an object and at 1 cm or less to this object. Data were analyzed with Any maze software (Stoelting Co, Wood Dale, IL, United States). The time of exploration was expression as a discrimination index (*T*_*N*_/(*T*_*F*_ + *T*_*N*_) × 100), where *T*_*N*_, exploration time for novel object; *T*_*F*_, exploration time for familiar object. Mice that explored objects less than 5 s were removed from analysis as before (Filali et al., 2009). All tests were done blind.

### Barnes Maze

Mice were acclimatized to the room 1 day before testing. Our Barnes Maze consisted of a white circular table with 20 holes evenly sized and spaced around the perimeter, as described before (Hernandez-Rapp et al., 2015). Spatial cues were placed in all the testing room with a homogeneous light (800 lux) and shrill sound (74 db). The training phase consisted of four trials for 4 days. During this phase, an escape box was placed within exit quadrant under one of the holes. The table was virtually divided in four quadrants: exit (with escape box), west, east, and opposite, with the same number of holes. The training phase ended when mice climbed into the escape box or when 180 s of exploration was reached. Upon the mice was entered in the escape box, the light and the sound were turn off and mice remained in this box during 60 s before returning it home cage. The localization of escape box and exit quadrant remained constant during all the trial and days. One (D5) and eight (D12) days following the last day of training (D4), memory was assessed in single 90 s probe trial test. During the probe trial tests, the escape box was removed with a false box identical to the other 19 holes. The speed, distance traveled, immobility time, time passed into each quadrant, primary time, and total time were measured with Any maze (Stoelting Co, Wood Dale, IL, United States). The primary and total error number, were measured manually. All tests were done blind.

### Immunofluorescence

Twenty-micrometer serial section of mouse half-brain sample were collected with cryotome at -20°C. Slices were incubated in citrate buffer (sodium citrate at 0.1 M and 10% ethanol, pH = 8.5) during 40 min at 70°C before 30 min at room temperature (RT). Then, slices were incubated in hydroxyperoxide during 30 min and sodium borohydrure 0.1% during 30 min. Slices were put in blocking solution (9% NBS, 1% BSA, and 0.5% Triton and PBS) during 1 h at RT. Finally, slices stayed over-night at 4°C in blocking solution with primary antibody: Iba1 (1:500, #019-19741, Wako), NeuN (1:500, MAB377, Millipore), MAP2 (1:500, AB5622, Millipore), or GFAP (1:500, clone SMI22, #835301, BioLegend). On the second day, the slices stayed 2 h at RT before an incubation of 1 h 30 min at RT in the secondary antibody solution (Alexa Fluor 568 anti-mouse #1736975, anti-rabbit #1832035, Alexa Fluor 488 anti-rabbit #A11034, anti-mouse #A11029) followed by 7 min in DAPI (D3571, ThermoFisher). Slices were then mounted on lamella and cover-slipped with Fluoromount mounting media (Invitrogen, Thermo Fisher Scientific; #00-4958-02). Slices were observed using a Zeiss AxioImager M2 microscope, zoom 10X/20X and images were processed with a computerized image analysis system (ZEN 2012 SP2 Software, Zeiss).

### ELISA

Endogenous murine Aβ40 and Aβ42 soluble peptides were measured by enzyme-linked immunosorbent assay (ELISA) (Life Technologies, catalog no. PP0812) following manufacturer’s instructions.

### Protein and RNA Extraction

Total proteins were extracted as previously described (Boscher et al., 2019). Frozen tissues were mechanically homogenized in 5× volume/weight of RIPA buffer (50 mM tris-HCL at pH 7.4, 150 nM NaCl, 1% NP-40, and 1 mM EDTA) supplemented with phosphatase inhibitor (1 mM activated sodium orthovanadate, 1 mM sodium fluoride), 1 mM phenylmethylsulfonyl fluoride, a complete mini EDTA-free protease inhibitor cocktail tablet (Roche Life Science), Triton 100X, and 0.5% sodium deoxycholate (Sigma, catalog n°D6750). Then tissues were lysed with a Sonic Dismembrator model 500 (Thermo Scientific). Lysates were incubated on ice for 20 min and centrifugated during 20 min at 20,000 × *g* at 4°C. The supernatant was removed and 15–20 μg of protein was mixed to the NuPAGE^®^ LDS sample buffer (Life Technologies) and 5% final volume of β-mercaptoethanol for Western blot analysis. Total RNA was extracted from cells using TRIzol reagent (Ambion by Life Technologies, catalog n°15596018) according to the manufacturer’s instructions.

### Real Time Quantitative RT-PCR

Pre-miRNA quantification were done using the miScript Precursor assays (Qiagen, n°218600) and QuantiTect^®^ SYBR Green PCR Master Mix (Qiagen, no1020722) following manufacturer’s instructions. Primers were purchased from Qiagen (pre-miR-138-2 ID: MP00004256; Snord95 ID: 00033726). MiR quantifications were done using the TaqMan miR Reverse Transcription Kit (Applied Biosystem, Burlington, ON, Canada) and TaqMan Universal Master Mix (Applied Biosystem, catalog no4324018) following manufacturer’s instructions. Primers were purchased from ThermoFisher (miR-138 ID: 000594; miR-99a: 000435; RNU19 ID: 001003). Mature miR-138 expression was normalized to RNU19 for brain tissue or miR-99a for cells. Pre-miR-138 expression was normalized to Snord95. The relative amounts of each transcript were calculated using the comparative Ct (2^–ΔΔ^*^*Ct*^*) method as before (Smith et al., 2011; Parsi et al., 2015).

### Western Blotting

Fifteen to twenty micrograms of protein were separated by TGX stain-free gel with Acrylamide 10% (Bio-Rad TGX Stain-Free FastCast Acrylamide Kit 10%) and transferred onto a 0.45 μm nitrocellulose membrane (Bio-Rad, Mississauga, ON, Canada) as previously described (Boscher et al., 2019). The membrane was blocked with 5% non-fat milk and 1% BSA (Bovine Serum Albumin, Bioshop, ALB007-500) then incubated at 4°C overnight with the appropriate primary antibodies: synaptophysin (1:1000, MAB5258-20UG, clone SY38, Millipore), PSD95 (1:1000, #2507, Cell Signaling), NeuN (1:1000, MAB377, Millipore), Iba1 (1:1000, #019-19741, Wako), GFAP (1:1000, clone SMI22, #835301, BioLegend), Bace1 (1:1000, D10E5, #5606S, Cell Signaling), Adam10 (1:1000, MAB946, R&D Systems), Aph1b/c (1:1000, gift of Bart De Strooper, KU Leuven, Belgium), Kindlin2 (Fermt2; 1:1000, ab74030, Abcam), Presenilin 1 (1:1000, AB5308, Millipore), Nicastrin (1:1000, PRS3983, Millipore), APP (1:1000, 6E10, sig-39320, Covance), GSK-3β (1:1000, 3D10, #9832, Cell Signaling), GSK-3S9 (1:1000, D2Y9Y, #14630, Cell Signaling), Tau total (1:40,000, #A0024, Dako), Tau PHF1 (1:1000, gift of Peter Davies, Albert Einstein University, New York, NY), Sirt1 (1:1000, H-300, sc-15404, Santa Cruz Biotechnology), AT8 (1:1000, MN1020, Thermo Scientific), and pS422 (1:1000, ab9664, Millipore). On the second day, membranes were incubated with secondary anti-IgG-HRP antibodies (1:5000, anti-mouse code 115-035-146, anti-rabbit code 111-035-144, Jackson ImmunoResearch) at RT for 1 h. The immune-reactive bands were acquired using Immobilon Western Chemiluminescent HRP Substrate (#WBKLS00500, Millipore) and visualized with the Fusion FX (Vilber Lourmat, Eberhardzell, Germany) imaging system. Normalization was performed on total proteins obtained via TGX stain-free gel kit following manufacturer’s instructions. Band intensities were quantified using the ImageJ software (Rueden et al., 2017).

### Statistical Analysis

Unless otherwise stated, all statistical analyses were performed using GraphPad Prism 7 Software (Graph Pad Software, Inc., La Jolla, CA, United States) as previously described (Boscher et al., 2019). Statistical differences were analyzed by the unpaired student *t*-test, column statistics test, one-way ANOVA or two-way ANOVA, and *p*-values < 0.05 were considered to be statistically significant.

## Results

### Generation of Wildtype Mice Overexpressing Pre-miR-138-2

**FIGURE 1:**
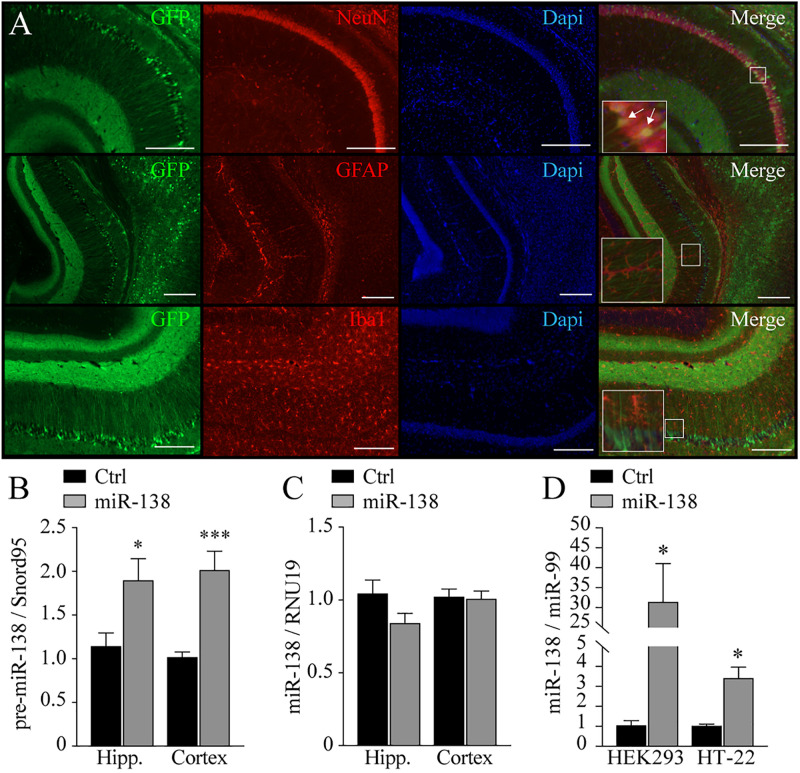
Expression and localization of AAV2 in the brain. **(A)** Characterization of AAV2/DJ8-CAG-eGFP in wild-type mice at 2 months of age: eGFP labeling in green; Neuronal marker: NeuN or Glial marker: GFAP or Iba1, in red. Nuclei marker: DAPI, in blue. Merge of green, red, and blue fluorescence. Immunofluorescence performed in half-brain of 2 months-old mice, 2 months after injections (*n* = 3). Note that most, if not all, co-stainings with eGFP are with the neuronal NeuN marker. Scale: 250 nm. **(B)** Expression of pre-miR-138 in hippocampus (Hipp.) and frontal cortex (Cortex) of mice at 4 months of age, injected with AAV2/DJ8-CAG-eGFP-MIR-138-2 (miR-138; *n* = 14) or AAV2/DJ8-CAG-eGFP (Ctrl; *n* = 16). **(C)** Expression of mature miR-138-5p in hippocampus (Hipp.) and frontal cortex (Cortex) of mice at 4 months of age injected with AAV2/DJ8-CAG-eGFP-MIR-138-2 (miR-138; *n* = 14) or AAV2/DJ8-CAG-eGFP (Ctrl; *n* = 16). **(D)** Expression of mature miR-138-5p in HEK293 and HT-22 cell lines (*n* = 3 in triplicate). Data presented as a mean ± SEM. *P < 0.05, **P < 0.01, ***P < 0.001 (unpaired student *t*-test).

To better understand the impact of increased MIR-138-2 gene dosage *in vivo*, we delivered the miR-138-2 precursor (pre-miR-138) in the newborn mouse brain using a modified AAV2 vector (AAV2/DJ8-CAG-eGFP) (Supplementary Figure S1; see also “Supplementary Methods” for details). This approach allows for widespread expression in the adult brain, especially in neurons but not microglia or astrocytes (Figure 1A). The choice to use wildtype mice in this study is to preserve all endogenous regulatory elements in genes (e.g., 3′-untranslated region) not commonly found in transgenic AD models expressing human (e.g., PSEN or APP) transgenes (Lu et al., 2019).

In 4-month-old adult mice injected with pre-miR-138 (namely, miR-138), we observed a significant increase of the miR-138 precursor in the hippocampus (1.9-fold, *p* = 0.01) and frontal cortex (twofold, *p* = 0.0005) (Figure 1B) when compared to an empty virus control (Ctrl). No significant changes in mature miR-138 levels were noted however (Figure 1C). These results are somewhat consistent with earlier studies suggesting a disparity between precursor and mature miR-138 levels in the mouse brain (Obernosterer et al., 2006; Schröder et al., 2014). We could nevertheless confirm that our virus construct could produce mature miR-138 levels in cultured cells (Figure 1D). We suspect therefore that mature miR-138 undergoes a strict regulation and/or a rapid turnover rate *in vivo*. A stabilization of miR-138 levels in adulthood (i.e., after 3–4 weeks of age) is consistent with this hypothesis (Liu et al., 2013; data not shown). Alternatively, ectopic mature miR-138 is below detection levels in whole tissues. Nevertheless, we could effectively develop an *in vivo* model recapitulating a mild (1.5- to 2-fold) increased gene dosage of pre-miR-138 (herein after referred to as miR-138).

### MiR-138 Overexpression Alters Behavior and Memory

**FIGURE 2:**
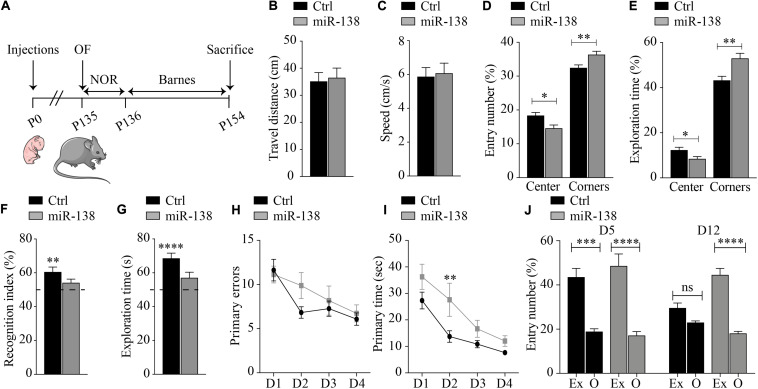
Alterations in learning, memory, and anxiety in miR-138 mice. **(A)** Experimental outline of the study herein. Open field: **(B)** travel distance, **(C)** speed, **(D)** entry number, and **(E)** exploration time into the center and the corners by miR-138 (*n* = 11) or Ctrl mice (*n* = 13). Data show with mean ± SEM: *P < 0.05, **P < 0.01, ***P < 0.001 (unpaired student *t*-test). Novel object recognition: **(F)** recognition index and **(G)** exploration time of the novel object by miR-138 and Ctrl mice. Data show difference from 50% with mean ± SEM: *P < 0.05, **P < 0.01, ***P < 0.001, ****P < 0.0001 (column statistics test). Barnes: **(H)** primary error and **(I)** primary time for miR-138 mice and Ctrl mice during the 4 days of testing (learning). Data shows in mean ± SEM: ns, non-significant; *P < 0.05, **P < 0.01 (two-way ANOVA). **(J)** Entry number in exit (Ex) versus mean of other (O) quadrants during the probe test at day 5 and 12 for Ctrl mice and miR-138 mice. All tests were performed on 30 mice in total (*n* = 13 males and *n* = 17 females). Data shows in mean ± SEM: ns, non-significant; *P < 0.05, **P < 0.01, ***P < 0.001, ****P < 0.0001 (Kruskal-Wallis test).

We next aimed to determine if miR-138 overexpression could influence behavior or cognition. For this, we used again 4-month-old mice injected with miR-138 or Ctrl AAV2 viruses at birth. No gross changes in animal basic behavior and phenotypes were observed during a SHIRPA assessment (Supplementary Table S1). We then performed, in sequence, an open-field (OF) test, a novel object recognition (NOR) test, and a Barnes Maze test as depicted in Figure 2A.

In the OF test, miR-138 expressing mice traveled the same distance and displayed the same speed than Ctrl mice (Figures 2B,C). We observed, however, that miR-138 mice had less entries into the center of the box than Ctrl mice (14.5% vs. 18.3%, *p* = 0.01), and entered more often in the corners than Ctrl mice (36% vs. 32.4%, *p* = 0.009) (Figure 2D). Similarly, miR-138 mice spent overall less time in the center of the box than Ctrl mice (8.3% vs. 12.2%, *p* = 0.03), and conversely spent more time in the corners than Ctrl mice (52.9% vs. 43.1%, *p* = 0.003) (Figure 2E). Thus, by spending more time in the corners, the miR-138 mice display an increased anxiety-like behavior.

In the NOR test, we observed that the miR-138 mice displayed differences in recognition index (miR-138: 54% vs. 50%, *p* = 0.10 and Ctrl: 60.4% vs. 50%, *p* = 0.004) and exploration time (miR-138: 56.9% vs. 50%, *p* = 0.06 and Ctrl: 68.5% vs. 50%, *p* < 0.0001) when compared to Ctrl mice (Figures 2F,G). Therefore, the miR-138 mice cannot differentiate familiar versus novel objects, unlike the Ctrl mice, likely due to an alteration of the non-spatial memory.

In the Barnes Maze, during the training phase, the primary error and primary time of Ctrl mice reached a plateau at day 2 (D2) and until day 4 (D4), as expected (Figures 2H,I). On the other hand, miR-138 mice displayed a significant increase of primary time at D2 compared to Ctrl mice (27.5 vs. 13.7 s, *p* = 0.005) (Figure 2I). During the probe phase, at D5, we saw that Ctrl mice entered more often in the exit quadrant (where escape box was previously hidden) compared to other quadrants (43.4% in exit vs. 18.8% mean of all others quadrant, *p* = 0.0003), as did the miR-138 mice (48.5% in exit vs. 17.1% mean of all others quadrant, *p* < 0.0001) (Figure 2J). In a second probe phase, at D12, Ctrl mice showed a similar number of entries in the exit quadrant compared to other quadrants (29.5% in exit vs. 22.9% mean of all other quadrants, *p* > 0.99). In contrast, however, miR-138 mice entered significantly more often in the exit quadrant compared to others (44.4% in exit vs. 18% mean of all other quadrants, *p* < 0.0001) (Figure 2J). Overall, these results suggest that miR-138 mice display subtle alterations in learning and memory retention. Collectively, these data are consistent with earlier findings (Lu et al., 2019) showing that miR-138 upregulation in mice causes alterations in learning and memory as well as anxiety-like behavior.

### Neuronal miR-138 Overexpression Promotes Aβ42 Production *in vivo*

**FIGURE 3:**
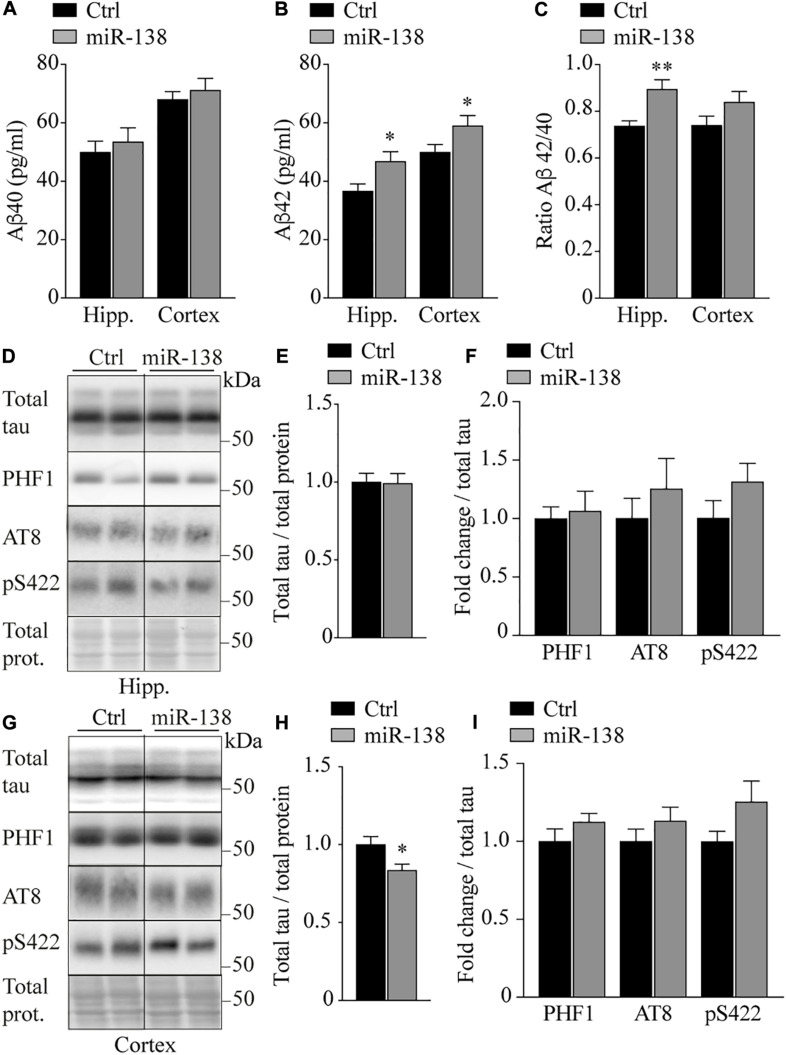
Increase of Aβ42 and decrease of total tau in miR-138 mice. ELISA of **(A)** endogenous murine Aβ40, **(B)** Aβ42, and **(C)** Aβ42/40 ratio from hippocampus (Hipp.) and frontal cortex (Cortex), in miR-138 mice (*n* = 11, sex mixed) compared to Ctrl mice (*n* = 13, sex mixed), 4 months after injection. **(D)** Representative Western blot of endogenous **(E)** total Tau and **(F)** PHF1, AT8, and pS422 quantifications in hippocampus (Hipp.). **(G)** Representative Western blot of endogenous **(H)** total Tau and **(I)** PHF1, AT8, and pS422 quantifications in frontal cortex (Cortex). Total tau bands were normalized to total proteins and phospho-tau bands were normalized to total tau. All analyses were performed on 30 mice in total (*n* = 13 males and *n* = 17 females). Data are presented as ±SEM: *P < 0.05 (unpaired student *t*-test).

Next, we sought to investigate if neuronal miR-138 overexpression *in vivo* could influence AD-related biological markers as seen in culture cells (Boscher et al., 2019). In miR-138-injected mice, we observed an increase in endogenous Aβ42 levels in the hippocampus (46.8 vs. 36.6 pg/ml, *p* = 0.02) and frontal cortex (59.0 vs. 49.9 pg/ml, *p* = 0.04) compared to Ctrl mice (Figure 3B). These effects were not seen with Aβ40 (Figure 3A), although we noticed an increase in the Aβ42/40 ratio in the hippocampus of miR-138 mice (0.89 vs. 0.73, *p* = 0.02) (Figure 3C). These results are in agreement with previous observations in AD mice (Lu et al., 2019). In the frontal cortex, we observed a small but significant decrease in total tau expression (0.83-fold, *p* = 0.02) in miR-138 compared to Ctrl mice (Figures 3G,H). However, no overall changes in tau phosphorylation (PHF1, AT8, and S422 phospho-epitopes) were noted in both regions (Figures 3D–I). Thus, miR-138 upregulation specifically affects Aβ42 in our mouse model with moderate effects on tau levels in one region tested.

**FIGURE 4:**
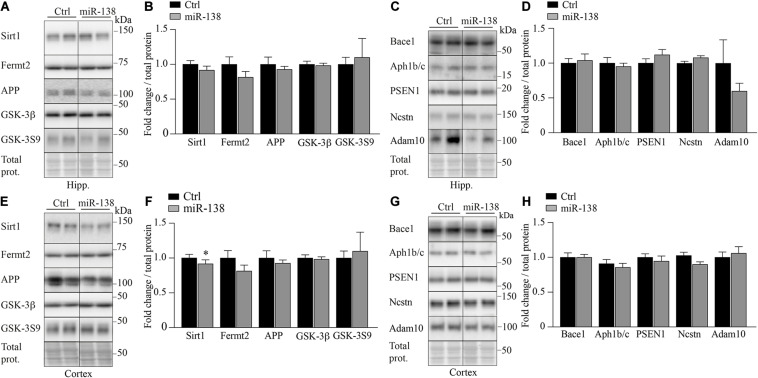
Decrease of Sirt1 in miR-138 mice. **(A)** Representative Western blot of endogenous **(B)** Sirt1, Fertm2, APP, GSK-3β, and GSK-3S9 quantification in hippocampus (Hipp.) of miR-138 mice compared to Ctrl mice, at 4 months of age. **(C)** Representative Western blot of endogenous **(D)** Bace1 Aph1b/c, PSEN1, Nicastrin (Ncstn), and Adam10 quantification in hippocampus (Hipp.) of miR-138 mice compared to Ctrl mice. **(E)** Representative Western blot of endogenous **(F)** Sirt1, Fertm2, APP GSK-3β, and GSK-3S9 quantification in frontal cortex (Cortex) of miR-138 mice compared to Ctrl mice, at 4 months of age. **(G)** Representative Western blot of endogenous **(H)** Bace1 Aph1b/c, PSEN1, Nicastrin (Ncstn), and Adam10 quantification in frontal cortex (Cortex) of miR-138 mice compared to Ctrl mice. Bands were normalized to total proteins. All analyses were performed on 30 mice in total (*n* = 13 males and *n* = 17 females). Data are presented as ±SEM: *P < 0.05, **P < 0.01, ***P < 0.001 (unpaired student *t*-test).

Previously, we have shown that miR-138 can regulate the expression of different genes involved in Aβ metabolism, including APP, Bace1, Fermt2 GSK-3β, and GSK-3β phosphorylated at Ser9 (pGSK-3S9) (Boscher et al., 2019). However, none of these proteins or epitopes were affected in miR-138 compared to Ctrl mice (Figures 4A,B,E,F). No changes were observed also in Presenilin 1, Nicastrin, and Aph-1b/c all major members of γ-secretase necessary for Aβ production in the brain (Figures 4C,D,G,H). Finally, a non-significant trend was seen for Adam10 (α-secretase) downregulation in the hippocampus (0.69-fold, *p* = 0.25) but not frontal cortex (Figures 4C,D,G,H).

We therefore turned toward bioinformatics in attempt to identify miR-138 targets known to influence Aβ and/or behavior. Our search using TargetScan^4^ revealed the direct miR-138 target Sirt1. In agreement with this, we observed a significant decrease of endogenous Sirt1 in the frontal cortex of miR-138 mice compared to Ctrl mice (0.74-fold, *p* = 0.03) (Figures 4E,F). The downregulation of Sirt1 is consistent the literature showing its effects on Aβ, memory, and anxiety (Gao et al., 2010; Libert et al., 2011; Hernandez-Rapp et al., 2016; Lu et al., 2019; Wahl et al., 2019). Sirt1 was also an effector candidate in AD mice overexpressing mature miR-138 (Lu et al., 2019).

### Changes in Synaptic and Inflammation Markers Following miR-138 Upregulation

**FIGURE 5:**
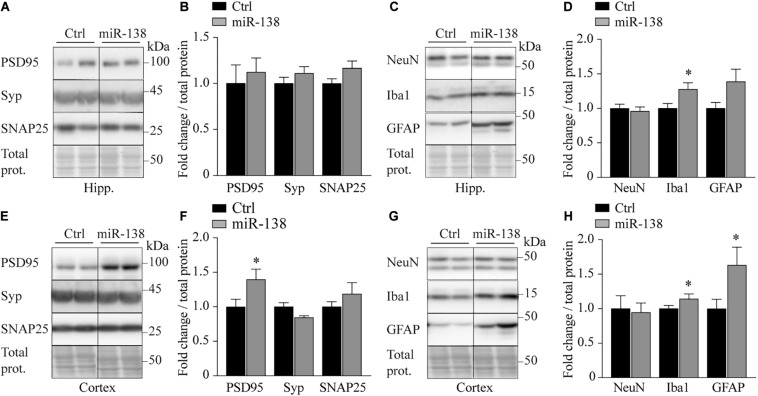
Abnormal brain integrity markers in 138 mice. **(A)** Representative western blot of endogenous **(B)** PSD95, synaptophysin (Syp), and SNAP25 quantification in hippocampus (Hipp.) of miR-138 mice compared to Ctrl mice, at 4 months of age. **(C)** Representative Western blot of endogenous **(D)** NeuN, Iba1, and GFAP quantification in hippocampus (Hipp.) of miR-138 mice compared to Ctrl mice. **(E)** Representative western blot of endogenous **(F)** PSD95, synaptophysin (Syp), and SNAP25 quantification in frontal cortex (Cortex) of miR-138 mice compared to Ctrl mice. **(G)** Representative Western blot of endogenous **(H)** NeuN, Iba1 and GFAP quantification in frontal cortex (Cortex) of miR-138 mice compared to Ctrl mice. Bands were normalized to total proteins. All analyses were performed on 30 mice in total (*n* = 13 males and *n* = 17 females). Data are presented as ±SEM: *P < 0.05, **P < 0.01, ***P < 0.001 (unpaired student *t*-test).

We finally sought to explore if miR-138 could impact brain integrity. We observed an increase of PSD95 (post-synaptic marker) (1.39-fold, *p* = 0.04) with a trend for decreased synaptophysin (pre-synaptic marker) (0.84-fold, *p* = 0.05) in the frontal cortex of miR-138 compared to Ctrl mice (Figures 5A,B,E,F). These results are consistent with the literature in AD patients (Leuba et al., 2008). We also noticed an increase of Iba1 (microglia marker) in the frontal cortex (1.16-fold, *p* = 0.02) and hippocampus (1.28-fold, *p* = 0.02) (Figures 5C,D,G,H). We lastly observed an increase of GFAP (astrocyte marker) in frontal cortex (1.39-fold, *p* = 0.02) with a trend toward an increase in the hippocampus (1.38-fold, *p* = 0.05) of miR-138 mice (Figures 5C,D,G,H). Taken together, these results suggest that a mild overexpression of pre-miR-138 impacts both neuronal and non-neuronal cells in the brain.

## Discussion

This study extends our previous research suggesting that increased MIR-138 gene dosage could influence AD risk (Boscher et al., 2019). The main findings of this study are threefold: first, we show that a mild overexpression of pre-miR-138 *in vivo* induces behavioral changes in non-transgenic wildtype mice, including learning, memory, and anxiety. These results are consistent with recent findings in AD mice overexpressing mutant human APP and PSEN1 (Lu et al., 2019). Secondly, miR-138 overexpression leads to an increase in soluble Aβ42, possibly via its effector target gene Sirt1 in the frontal cortex, again in agreement with earlier observations (Lu et al., 2019). The increase of Aβ42 in the hippocampus, however, could rely on independent or more transient mechanisms. Thirdly, we noticed changes in brain integrity markers, including PSD95, Iba1, GFAP, and Synaptophysin, as seen in vulnerable regions in AD brain in humans (Braak and Braak, 1991; Leuba et al., 2008). In sum, our findings *in vivo* in non-transgenic mice agree with the proposed role of miR-138 in synapses (Siegel et al., 2009), inflammation (Wei et al., 2016; Wang M. et al., 2019; Wang Y.Q. et al., 2019), as well as memory/learning and anxiety (Schröder et al., 2014; Li et al., 2018).

The observation that miR-138 upregulation can specifically influence Aβ42 (and Aβ42/Aβ40 ratios) is noteworthy and fully consistent with the effects of miR-138 on human Aβ42 recently seen in APP/PS1 mice (Lu et al., 2019). It is well known that human Aβ42 is more aggregation-prone and neurotoxic than Aβ40 (Jarrett et al., 1993; Iwatsubo et al., 1994; Kim et al., 2007). The increase of endogenous Aβ42 observed in miR-138-overexpressing wildtype mice, albeit not promoting Aβ plaques *per se*, could alter synaptic activity in the hippocampus and/or cortex and hence influence cognition locally or at distance as documented herein (Furukawa et al., 1996; Hwang et al., 2002; Kamenetz et al., 2003; Priller et al., 2006). Indeed, Aβ42 is also known to act in, or promote, inflammation (Soscia et al., 2010) as well as impair learning and memory (Caccamo et al., 2010; Faucher et al., 2016), among other behaviors. This idea is consistent with the changes observed on different brain integrity markers. Note that alterations in synaptic markers and function were observed also by Lu et al. in APP/PS1 mice following miR-138 overexpression. This latter group observed also impaired memory following miR-138 overexpression in a Water Maze. While the effects we observed herein in the Barnes Maze are more subtle, we suspect that specific memory-related functions go awry such as retention (e.g., the miR-138 mice could not remember that the escape hole had disappeared). These results are nevertheless consistent with the alterations in memory in the NOR test and overall loss of cognitive abilities following miR-138 overexpression seen also by Lu et al. As shown herein, the overall subtle effects on behavior and pathologies could be attributed to the low or undetectable levels of ectopic mature miR-138. This could be caused by, at least in part, the AAV injections at P0 (necessary to achieve widespread brain expression) combined with the strict regulation of miR-138 soon after birth to achieve a peak in adulthood. Further tests are now required to determine the precise levels learning and memory that are affected by miR-138 overexpression (e.g., spatial vs. non-spatial memory), using perhaps also alternative delivery strategies such as stereotactic injections of lentiviruses (Li et al., 2020).

Precisely how miR-138 alters Aβ42 remains unclear. The search for direct targets of miR-138 lead us to focus on Sirt1, known to play a role in modulating Aβ and importantly, behavior such memory and anxiety in mice (Gao et al., 2010; Libert et al., 2011; Hernandez-Rapp et al., 2016; Lu et al., 2019). Lu et al. also proposed Sirt1 as main effector of Aβ and behavior alterations in AD mice overexpressing miR-138. While promising, more experiments are required to determine if additional miR-138 targets (direct or indirect) can be involved in Aβ modulation and associated phenotypes. Interestingly, Lu et al. observed a downregulation of ADAM10 in their model, which could explain also in part the specific effects on Aβ42. In our hands, ADAM10 levels were not consistently changed but other mechanisms of actions (e.g., shedding activity) cannot be excluded. Another miR-138 target previously involved in AD, retinoic acid receptor α, was below detection levels in our brain samples (Boscher E, unpublished observation). Yet, other candidates such as GABRA6, a miR-138 target that is associated to anxiety (Muiños-Gimeno et al., 2011), combined with specific gene knockouts or miR-138 seed mutagenesis can address this and other issues in the future.

In contrast to our previous studies in cultured cells *in vitro* (Boscher et al., 2019), we observed no significant changes in tau phosphorylation following miR-138 overexpression *in vivo*. This is perhaps not surprising given that tau hyperphosphorylation often occurs at much later ages in AD transgenic mice (Andorfer et al., 2003; Oddo et al., 2003). Whether similar age-related effects occur in our mouse model needs to be tested. Indeed, the choice to use young (4-month-old) mice in this pilot study was based on several factors (e.g., increased brain plasticity, AAV system) but, admittedly, is associated with age-related and other limitations. Interestingly, total tau expression was slightly lower in miR-138 mice. We hypothesize that such effects are due to synaptic remodeling, given the localization and function of tau at synapses, and overall changes in synaptic markers and behavior. Future studies are required to determine the long-term effects of miR-138 on tau metabolism *in vivo* using possibly human tau that is more prone to abnormal hyperphosphorylation and aggregation. The overall effects of stronger miR-138 expression on tau and behavior need also to be addressed.

In sum, this study extends and confirms recent findings linking increased miR-138 dosage and AD risk in a more physiological context. It opens the door to better understand the importance of CNVs in miR genes in AD and other brain disorders.

## Data Availability Statement

The raw data supporting the conclusions of this article will be made available by the authors, without undue reservation.

## Ethics Statement

The animal study was reviewed and approved by VRRC Comité de protection des animaux.

## Author Contributions

EB designed, planned, and performed the experiments. CG helped with experimental planning and rodent experiments. CG, SP, RK, and AL provided valuable input to experimental planning, and contributed to scientific discussions. EP provided valuable material and scientific input. SH designed, planned, and supervised the experiments. SH and EB wrote the article. All authors contributed to the article and approved the submitted version.

## Conflict of Interest

The authors declare that the research was conducted in the absence of any commercial or financial relationships that could be construed as a potential conflict of interest.

## Footnotes

1 https://cervo.ulaval.ca/fr/plateforme-doutils-moleculaires-pom/

2 http://genome.ucsc.edu/

3 https://www.jax.org

4 http://www.targetscan.org/
