# Supplementary material for: MicroRNA-138 Overexpression Alters Aβ42 Levels and Behavior in Wildtype Mice
Source: Front Neurosci. 2021 Jan 14;14:591138. doi: 10.3389/fnins.2020.591138 (PMC7840584; doi:10.3389/fnins.2020.591138)
Supplement: Supplementary file 1 [file Data_Sheet_1.PDF]

Supplementary Material

1 Supplementary Methods

1.1 SHIRPA

SHIRPA (SmithKline Beecham, Harwell, Imperial College, Royal London Hospital, phenotype assessment) was performed in a Perspex clear plastic box (55 cm x 33 cm x 18 cm) as previously described (Filali et al., 2009). In brief, we analyzed the body and shape (color and aspect of coat, head morphology, aspect of teeth, eyes, whiskers and tail) as well as body movement (repeat jumps, tremor, breathing, locomotion). Mice were also put on metal grid to analyze mice motion and grip. The grid was turned at 90° and 180° to study muscular capacity. A sound of 90 decibels was delivered to study audition capacity (jump, freezing, none reaction). Then, mice were put on a wire to observe balance. Finally, we looked for status epilepticus, abnormal jumping, and aggressive/stressed behavior.

2 Supplementary Figures and Tables

2.1 Supplementary Figure 1

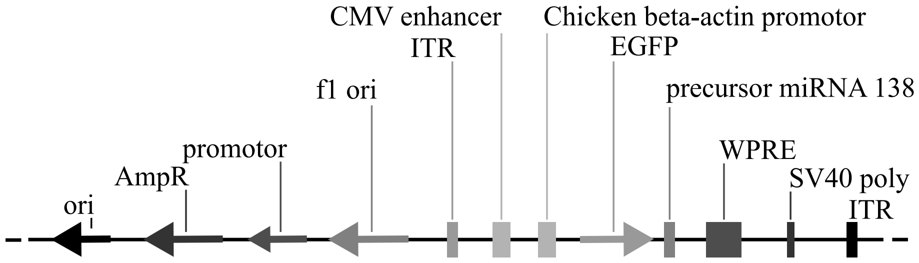

Schematic representation (not to scale) of the AAV2/DJ8-CAG-eGFP plasmid construct used in this study. One-hundred and fifty basepairs (bp) upstream and downstream of mmu-pre-mir-138-2 sequence was cloned into the AAV2 construct after the eGFP stop codon.

2.2 Supplementary Figure 2

mmu-miR-138-2
5′ gguugcugC**AGCUGGUGUUGUGAAUCAGGCCG**ACGAGCAGCGCAUCCUCUUACCCGGCUAUUUCACGACACCAGGGUUGcaccc

hsa-miR-138-2
5′ CGUUGCUGC**AGCUGGUGUUGUGAAUCAGGCCG**ACGAGCAGCGCAUCCUCUUACCCGGCUAUUUCACGACACCAGGGUUGCAUCA

Sequence alignment between mouse and human pre-miR-138 (stem-loop sequence based on miRbase.org). The miR-138-5p mature sequence (in bold) is 100% conserved. Note that the human precursor sequence is 14 bp longer according to miRbase.org.

2.3 Supplementary Table 1

SHIRPA primary screen in miR-138 and WT mice			
Tests		Ctrl (n= 13)	miR-138 (n= 11)
Viewing jar			
Body position	Standing	100%	100%
Spontaneous activity	Inactive	0%	0%
	Active	100%	100%
	Excessively	0%	0%
Respiration rate	Normal	100%	100%
	Accelerated breathing	0%	0%
Body tremor	None	100%	100%
Coat color	Normal : black	100%	100%
Head morphology	Abnormal	0%	0%
Kinky tail	Present	0%	0%
Whiskers	Missing	0%	0%
	Short	0%	0%
Lacrimation	None	100%	100%
Salivation	None	100%	100%
Eye	Normal	100%	100%
	Mild abnormalities	0%	0%
	Severe abnormalities	0%	0%
Piloerection	Normal	100%	100%
Open-field			
Gait	Normal	100%	100%
Horizontal grid			
Paw grasping	Present	100%	100%
Grip strength	Active	100%	100%
	Moderate	0%	0%
	Slight	0%	0%
Horizontal bar			
Wire maneuver	Active	100%	100%
	Difficulty	0%	0%
Vertical grid			
Wire maneuver			
	Climb	100%	100%
	Fall	0%	0%
Handling-related behaviors			
Agression	Present	0%	0%
Fear	Present	0%	0%
Vocalization	Present	0%	0%
Body measurement			
Weight (g)	Males	30,98 (± 3,38)	31,21 (± 2,63)
	Females	24,23 (± 1,77)	26,08 (± 4.14)
Temperature (°C)	Males	36,7 (± 0,98)	36,62 (± 0,56)
Age			
Mean of age (day)		131,4 (± 3,4)	132,2 (± 2,9)
Gender			
Males		38% (n= 5)	64% (n= 7)
Females		62% (n= 8)	36% (n= 4)

Overview of the SHIRPA analysis of both control (n= 13) and miR-138 (n= 11) mice injected with AAV2 viruses. Tests were performed when mice were aged 4 months-old, prior to sacrifice. No differences were observed between groups of mice.
